# NAFLD-Related Hepatocarcinoma: The Malignant Side of Metabolic Syndrome

**DOI:** 10.3390/cells10082034

**Published:** 2021-08-09

**Authors:** Anna Michelotti, Marco de Scordilli, Lorenza Palmero, Michela Guardascione, Mario Masala, Rossana Roncato, Luisa Foltran, Elena Ongaro, Fabio Puglisi

**Affiliations:** 1 Centro di Riferimento Oncologico di Aviano (CRO), Department of Medical Oncology, IRCCS, 33081 Aviano, Italy; anna.michelotti@cro.it (A.M.); marco.descordilli@cro.it (M.d.S.); lorenza.palmero@cro.it (L.P.); mario.masala@cro.it (M.M.); luisa.foltran@cro.it (L.F.); fabio.puglisi@cro.it (F.P.); 2 Department of Medicine (DAME), University of Udine, 33100 Udine, Italy; 3 Experimental and Clinical Pharmacology Unit, Centro di Riferimento Oncologico di Aviano (CRO), IRCCS, 33081 Aviano, Italy; michela.guardascione@cro.it (M.G.); rroncato@cro.it (R.R.)

**Keywords:** HCC, hepatocellular carcinoma, NAFLD, NASH, metabolic syndrome, insulin resistance

## Abstract

Hepatocellular carcinoma (HCC) is the seventh most common cancer worldwide and the second leading cause of cancer-related mortality. HCC typically arises within a cirrhotic liver, but in about 20% of cases occurs in absence of cirrhosis. Among non-cirrhotic risk factors, non-alcoholic fatty liver disease (NAFLD) currently represents the most important emerging cause of HCC in developed countries. It has been estimated that annual incidence of HCC among patients with non-cirrhotic NAFLD is approximately 0.1–1.3 per 1000 patients/year and ranges from 0.5% to 2.6% among patients with non-alcoholic steatohepatitis (NASH) cirrhosis. However, only a few clinical trials enrolling HCC patients actually distinguished NAFLD/NASH-related cases from other non-cirrhotic causes and therefore evidence is still lacking in this subset of patients. This review aims to describe the biology underpinning NAFLD development, to investigate the main molecular pathways involved in its progression to NASH and HCC and to describe how different pathogenetic mechanisms underlying the onset of HCC can have an impact in clinical practice. We hereby also provide an overview of current HCC treatment options, with a particular focus on the available data on NAFLD-related cases in practice-changing clinical trials.

## 1. Introduction

Hepatocellular carcinoma (HCC) is the seventh most common cancer worldwide and the second leading cause of cancer-related mortality, with substantial differences across geographical areas due to the different prevalence of risk factors [1]. 

HCC typically arises within a cirrhotic liver, but about 20% of cases occurs within a non-cirrhotic liver [2]. 

Main risk factors for HCC are represented by the presence of viral hepatitis due to hepatitis B virus (HBV) or hepatitis C virus (HCV) infection [3,4]. HBV is an enveloped hepatotropic DNA virus, belonging to the Hepadnaviridae family, that replicates by reverse transcription of an RNA pregenome, integrating into the host’s genome and leading over time to the onset of mutations in the hepatocytes, a process that underlies the progression of carcinogenesis [5]. The risk of developing HCC for patients with chronic HBV infection is estimated between 10 and 25% [6]. Antiviral therapy and vaccination programs are two key elements to reduce the incidence of HBV-related HCC [6,7]. Chronic HCV infection is also associated with an increased risk of HCC, 10- to 20-fold higher compared to the general population [5]. HCV virus is a single stranded RNA virus, belonging to Flaviviridae family, that does not integrate its own genome into the host cell’s genome; in this case, the basis of carcinogenesis is chronic inflammation, which progressively leads to fibrosis and subsequently, in about 90% of the cases resulting in HCC, to cirrhosis [8]. Therefore, incidence and prevalence of HCC are higher in Africa and Asia compared to Western countries, due to the higher incidence of hepatotropic viral infections [1,7].

Apart from cases linked to hepatotropic viral infections, liver cirrhosis from other causes represents the main cause of HCC, acting synergically with HBV and HCV chronic infections, HIV infection, other hepatotropic viral infections, diabetes and chronic alterations of transaminase blood levels.

Other known risk factors for HCC are alcohol abuse and some inherited metabolic diseases like hemochromatosis, characterized by the accumulation of iron in hepatocytes, alpha-1 antitrypsin deficiency, with the accumulation of abnormal alpha-1 antitrypsin, Wilson’s disease, characterized by copper deposits in hepatocytes, and tyrosinemia type I, characterized by abnormal accumulation of tyrosine and its metabolites in the liver, kidney and central nervous system.

Among non-cirrhotic conditions, non-alcoholic fatty liver disease (NAFLD) deserves a specific mention. This condition refers to the presence of hepatic steatosis, with or without inflammation or fibrosis, in absence of other causes for secondary liver fat accumulation (e.g., alcohol consumption). Major risk factors include obesity, type 2 diabetes mellitus and dyslipidemia; therefore, it can be considered as the hepatic expression of metabolic syndrome. The relationship between NAFLD and metabolic dysfunctions has recently led to the new term ‘MAFLD’ (metabolic associated fatty liver disease), that underlines the coexistence of hepatic steatosis and metabolic dysfunctions [9]. Diagnostic criteria of MAFLD are based on a histological, radiological or laboratory evidence of liver fat accumulation (hepatic steatosis) in both young and adult patients of at least 16 years old, in addition to one of the following criteria: overweight/obesity, type 2 diabetes mellitus or evidence of metabolic dysregulation [10,11].

NAFLD currently is the most important emerging cause of HCC in non-cirrhotic liver in developed countries [12], in which it represents the most common cause of chronic liver disease affecting about a quarter of the general population [13,14]. The histologic spectrum of NAFLD ranges from macrovesicular steatosis to hepatic inflammation and fibrosis, known as non-alcoholic steatohepatitis (NASH). For reasons not yet fully understood, NAFLD is more likely to develop in men than in women, with an incidence peak in the sixth decade of life [15]. In the United States, prevalence of this condition can be estimated at around 30–40% in men and 15–20% in women, and is even higher in patients with type 2 diabetes mellitus, with a reported incidence of 42.6–69.5% in large samples of type 2 diabetic patients [16,17]. Among individuals with NAFLD, about 20–30% develops NASH, which then progresses to cirrhosis in 10–20% of cases [7]. There is evidence that a significant proportion of HCC cases in NAFLD patients (about 20–30%) arises in association with non-cirrhotic liver, however, due to the low risk (<1% per year) of developing HCC in this subpopulation of patients, surveillance in this subgroup is not considered cost-effective [18,19,20]. A separate case seems to be represented by patients with NAFLD and diabetes mellitus: the latter appears as the most important independent risk factor for the occurrence of HCC in NAFLD patients [21]. Although liver biopsy remains the gold standard, the initial diagnosis of NAFLD is usually radiological in daily clinical practice and the condition is diagnosed using imaging techniques detecting the presence of an accumulation of fat in the liver that involves at least 5% of the organ [22]. The most used imaging technique is abdominal ultrasound, with good accuracy and relative ease of performance; the most accurate imaging tool, but not routinely performed, is abdominal magnetic resonance (MRI). Currently, the cornerstone of NAFLD treatment is the control of insulin resistance in conjunction with weight loss and lifestyle modification: reduction of caloric intake, high fiber and low red meat diet, animal fats and refined sugars intake; and an active lifestyle with daily physical activity [23].

Drawing from these considerations, this review aims to describe the main molecular mechanisms underlying the development of NAFLD and its progression to HCC, not necessarily through the classical progression from fibrosis to cirrhosis to HCC. Moreover, the main purpose was also to investigate how pathogenetic mechanisms underlying HCC onset can have an impact in clinical practice in terms of surveillance, diagnosis and possible differences in the therapeutic approach.

## 2. Understanding Molecular Pathways Underlying NAFLD

**Figure 1:**
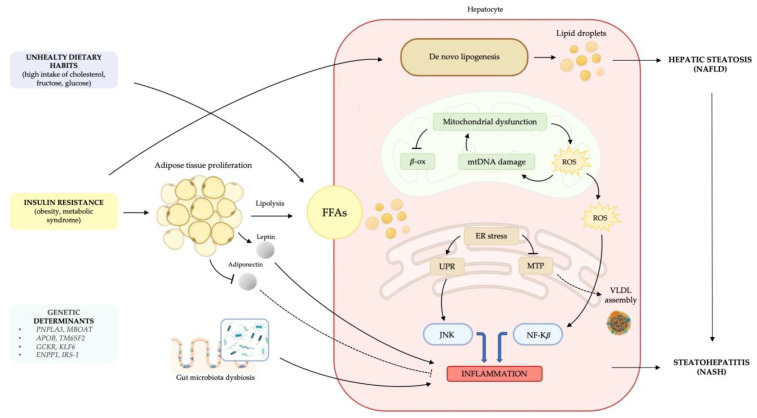
Pathogenesis of non-alcoholic fatty liver disease. The complex interplay between environmental, genetic, and metabolic factors results in an imbalance between intrahepatic lipid retention and disposal. Increased FFAs uptake derives from both high fat diet and adipose tissue lipolysis. Peripheral insulin resistance promotes adipose tissue proliferation and adipocyte disfunction, induces lipolysis and release of adipokines, namely leptin and adiponectin with pro-inflammatory effects. Insulin resistance also determines an increase in hepatic de novo lipogenesis, worsening lipid liver accumulation. Toxic lipid species cause mitochondrial dysfunction, oxidative stress with ROS production and ER stress leading to the activation of UPR response. All these processes are involved in the activation of an inflammatory response through JNK e and NF-κβ pathways. Gut microbiota dysbiosis is also implicated in hepatotoxic oxidative damage. Abbreviations: FFAs, free fatty acids; β-ox, beta oxidation; mtDNA, mitochondrial DNA; ROS, reactive oxygen species; ER, endoplasmic reticulum; UPR, unfolded protein response; MTP, microsomal triglyceride transfer protein; JNK, c-Jun N-terminal kinase; NF-Kβ, nuclear factor kappa-light-chain-enhancer of activated B cells; VLDL, very low-density lipoprotein; NAFLD, non-alcoholic fatty liver disease; NASH, non-alcoholic steatohepatitis.

NAFLD is a clinicopathologic entity encompassing a broad spectrum of hepatic dysfunctions, ranging from simple hepatic steatosis secondary to excessive lipid accumulation (NAFL) to necroinflammation and steatohepatitis (NASH). Despite research efforts, the molecular basis of the disease is still unclear. Most widely accepted theories initially speculated on a “two-hits model”, in which the first hit is triggered by hepatic lipid accumulation secondary to a sedentary lifestyle, obesity, and insulin resistance, leading to an increased susceptibility of liver tissue to damaging factors that constitutes the second hit responsible for clinical manifestation [24]. Over the years, this simplistic hypothesis has been overcome by a more complex paradigm that includes the synergic effect of “parallel, multiple-hits”, in conjunction with environmental and genetic factors in predisposed individuals [25]. The multiple-hits model offers a more detailed explanation of the metabolic and molecular changes characteristic of NAFLD development and progression (Figure 1).

### 2.1. Metabolic Dysfunction: Lipid Accumulation, Lipotoxicity and Insulin Resistance 

A key role in NAFLD pathogenesis is played by metabolic dysregulation, as a consequence of insulin resistance and excessive accumulation of hepatic lipids, mainly in the form of triglycerides (TG) [26]. Recent evidence suggests that the total amount of TG is not the main determinant of lipotoxicity. The currently accepted theory is that free fatty acids (FFAs), rather than TG, are the main factors responsible for inflammatory liver injury [27]. Hepatic metabolism of FFAs produces specific lipid toxic substances that act as damaging agents on hepatocytes and affect cellular metabolism via multiple signaling cascades involving inflammation and oxidative stress. Moreover, ceramides, composed by sphingosine and a fatty acid, are a family of lipid molecules implicated in several crucial mechanisms of NAFLD pathogenesis. This lipid class, in its free unconjugated form, exerts a direct lipotoxic effect triggering hepatocytes’ cell death and promoting inflammation via interaction with TNFα [28]. Ceramide production has also been linked to weight gain, glucose intolerance and insulin resistance [29]. Thus, steatosis can be viewed as an epiphenomenon of altered lipid metabolism and an early adaptive response to hepatocyte damage caused by disturbed FFAs balance [27]. Hepatic steatosis reflects the disturbance of lipid metabolism, due to an increased import from adipose tissue or a decreased hepatic export of FFAs. Excessive liver uptake of FFAs is due to an increased circulating pool of non-esterified fatty acids absorbed by the liver in proportion to their serum concentration, and to the excessive conversion of carbohydrates and proteins to TG [30]. NAFLD patients, in fact, typically have unhealthy dietary habits with high consumption of esterified fats, with saturated FFAs and cholesterol. This condition is strongly associated with obesity, hypercaloric diet and overeating, leading to insulin resistance and impaired lipid metabolism. Of note, fatty liver can also occur in lean individuals who share metabolic characteristics with obese patients, such as insulin resistance and dyslipidemia, which is referred to as “metabolically obese-normal weighted patients”, in whom environmental components and genetic predisposition have a strong influence [31]. Liver FFAs originate either from the diet or from adipose tissue via lipolysis and de novo lipogenesis in the liver. Once in hepatocytes, FFAs metabolites can enter esterification or beta-oxidation pathways. Consequently, impaired mitochondrial beta-oxidation of FFAs to ATP [32], together with the inhibition of TG incorporation into very low-density lipoprotein (VLDL) secretion by blocking microsomal triglyceride transfer protein (MTP), promotes hepatic lipid disposal [30,33]. Hepatic de novo lipogenesis is regulated by several transcription factors, such as sterol regulatory element-binding protein-1 (SREBP-1), carbohydrate response element-binding protein (ChREBP), and peroxisome proliferator-activated receptor (PPAR)-γ, which are regulated by insulin levels and are involved in cell cholesterol homeostasis [34,35]. The state of insulin resistance, induced by metabolic syndrome, obesity or genetic predisposition, is a well-established cardinal feature of NAFLD [36,37], as it leads to important alterations in lipid metabolism. These include increased lipolysis due to proliferation and dysfunction of peripheral adipose tissue, increased TG synthesis, and consequent excessive hepatic uptake of FFAs. In addition, adipose tissue is actively involved in this process through its endocrine functions. Obesity-related adipocyte hypertrophy and insulin resistance cause an imbalance in adipose tissue hormone secretion of adipokines, such as leptin and adiponectin. In particular, adiponectin, present in lower levels in NASH patients [38], has liver-protective functions by enhancing lipid clearance from plasma, stimulating beta-oxidation of FFAs, and also has anti-inflammatory effects by inhibiting the release of pro-inflammatory cytokines, such as TNFα and IL-6 [39]. Leptin, whose serum levels are higher in obese individuals, has opposite effects by stimulating the maintenance of a low-grade pro-inflammatory state and by having a pro-fibrogenic effect on the liver [40,41] through the activation of hepatic stellate cells (HSCs) [42].

### 2.2. The Onset of a Chronic Inflammatory State

Accumulation of TG and toxic lipid species in the liver leads to structural and functional alterations in mitochondrial function, resulting in severe impairment of fat homeostasis, respiratory chain deficiency, and overproduction of hepatotoxic oxygen free radical species (ROS) [43]. The presence of ROS in an environment enriched in lipids induces lipid peroxidation, which in turn leads to further damage to the respiratory chain, creating a vicious cycle. All these molecules are also involved in the activation of inflammatory pathways by producing various pro-inflammatory cytokines (TNFα, TGF-β) [44]. Changes in redox status or excessive protein synthesis also alter the folding capacity of the endoplasmic reticulum (ER) and activate the so-called “unfolded protein response” (UPR), an adaptive response that aims to restore homeostatic balance. ER stress indirectly affects TG accumulation in the liver by promoting insulin resistance. Furthermore, UPR in NAFLD promotes the activation of c-jun terminal kinase (JNK) and nuclear factor kappa-beta (NF-κB), which are actively involved in the hepatocyte inflammatory process and apoptosis [45]. Recent studies have provided evidence that gut microbiota is involved in hepatotoxic oxidative damage and that its specific composition may play a role in both inflammatory and fibrotic responses in NAFLD patients [26]. The persistent inflammatory stimulus combined with the constant exposure of hepatocytes to oxidative damage represents the key to NAFLD progression to NASH and potentially to carcinogenesis.

### 2.3. Genetic Predisposition

A hallmark of NAFLD is the substantial inter-patient variation in disease phenotype and progression. Genetic variability is currently considered one of the most important and promising areas. The heterogeneity of the disease phenotype and progression is explained by the complex interaction between the initial liver insult and both environmental and host genetic predisposition [46]. Genome Wide Association Studies (GWAS) identified several gene polymorphisms that may influence all pathophysiological levels of the disease: onset, severity and rate of progression, and response to treatment. The most studied genes are those involved in insulin resistance, lipid and glucose metabolism, oxidative stress, renin-angiotensin system, immune regulation, and fibrosis. These findings have led many researchers to focus on genetic factors that may play a role in the etiology of NAFLD [47]. Regardless, the impact of genetic polymorphisms on NAFLD pathophysiology differs between ethnic groups [48]. Findings with clinical relevance to the Caucasian population are summarized below. Twin studies provided evidence that hepatic steatosis and fibrosis, key early events in NAFLD pathogenesis, have a strong hereditary component [49]. Several studies have identified genetic variants associated with altered hepatic lipid metabolism through the impairment of VLDL secretion (*APOB*, *TM6SF2* rs58542926 C>T) [50], increased storage of lipid droplets (*PNPLA3* rs738409 C>G, *MBOAT*) [51,52], and regulation of de novo lipogenesis (*GCKR* rs780094 A>G, *KLF6* rs3750861 G>A) [53] to be associated with NAFLD. Also some polymorphisms (rs1800591 or -493G>T and rs3816873) in the microsomal triglyceride transfer protein gene, *MTTP*, have been associated with NAFLD. NASH patients with the -493GG genotype showed a more atherogenic postprandial lipid profile compared with the other genotypes [54]. A recent meta-analysis confirmed the correlation with NASH of the G allele for this polymorphism [55]. In addition, polymorphisms of *ENPP1* and *IRS-1*, which are involved in hepatic insulin signaling, are associated with decreased insulin receptor activity and severe fibrosis in NAFLD patients [56]. Polymorphisms (45TT, 276GT/TT) in the gene encoding adiponectin, involved in hepatic and peripheral glucose metabolism, independently predict liver disease severity in NASH by hepatic steatosis and necro-inflammatory grade along with postprandial adiponectin levels [38]. Other variants appear to have a protective role in the development of NAFLD/NASH. This is the case with the rs72613567 variant of hydroxysteroid 17-beta dehydrogenase 13 (HSD17B13), a liver protein involved in lipid metabolism. This loss-of-function variant reduces the risk of developing NAFLD, non-alcoholic cirrhosis [57] and also HCC, even with an alcoholic etiology [58].

In this scenario, the increasingly available high-throughput gene sequencing technologies will soon provide new insights in this field.

## 3. From NAFLD to HCC, a Roadmap Not So Winding

The role of NAFLD and metabolic dysregulation in the etiology of HCC is underestimated by current epidemiologic data and is expected to increase in the coming decades with the increasing incidence of obesity and diabetes. NASH is considered the third most common cause of HCC worldwide and the relative incidence is rapidly increasing at a rate of approximately 10% annually [59].

HCC from cryptogenic etiology is a recognized entity in literature, ranging from 6.9% to 50% [60]; in particular, in patients with HCC originating from cryptogenic cirrhosis, metabolic syndrome usually has higher prevalence; metabolic dysregulation and NASH could explain tumorigenesis, at least in part, in this pool of patients [61]. Some authors suggest that cryptogenic HCC may be a long-term evolution of NAFLD in a large proportion of cases, in which fatty deposits in the liver have been depleted, while other systemic signs of the metabolic syndrome are still evident [62]. Moreover, NAFLD and metabolic syndrome are also associated with a higher incidence of HCC independently from a cirrhotic evolution [63]. The process leading from NAFLD and NASH, with or without cirrhotic liver, to HCC is a continuum. Many factors can potentially contribute to HCC pathogenesis, from activation of systemic and local inflammatory and immune pathways to the metabolic changes and direct lipotoxicity associated with metabolic syndrome and insulin resistance, to the possible role of alterations in the gut bacterial flora or genetic predisposition. HCC carcinogenesis in chronic liver disease proceeds through a dysplasia-carcinoma sequence with multiple oncogenic mechanisms. Pre-neoplastic evolution consists of chronic hepatitis, cirrhosis, or both, a period during which the disruption of cell homeostatic mechanisms leads to irreversible genomic alterations [64]. In NASH, hepatocytes with microvesicular steatosis express more inflammatory markers (e.g., ICAM-1) than normal and consequently recruit a larger amount of phlogosis-regulating cells (e.g., macrophages, regulatory T cells) [65]. Alterations in the microenvironment also lead to loss of liver macrophages and Kupffer cells. Moreover, iron metabolism is altered in the development of NAFLD/NASH, with excessive iron deposition that further increases chronic inflammatory stress, promotes the production of ROS and activates immune cells [66]. 

Another recently discovered player in the progression from NASH to HCC is fibroblast growth factor 21 (FGF21), which is normally expressed in hepatocytes, reduces fat deposition in the liver and acts as an inflammation suppressor. Studies have found that it plays a key role in lowering IL-17A levels and thus NASH development and transition to HCC [67].

In patients with NAFLD and/or metabolic syndrome, lipid storage in the liver is increased by several mechanisms, such as increased hepatic lipogenesis, increased portal delivery from peripheral storage areas, and decreased elimination by fatty acid oxidation [68]. Lipotoxicity due to excessive lipid accumulation causes chronic inflammatory stress, with the production of ROS and alteration of normal cellular functions. Moreover, dysmetabolic syndrome also leads to the development of systemic insulin resistance, with compensatory hyperinsulinemia and with evidence that insulin and insulin-like growth factor (IGF) may directly contribute to the development of liver cancer through various carcinogenic pathways [69].

Another mechanism associated with the development of HCC is alteration of the gut microbiota. In obesity/metabolic syndrome, higher systemic levels of lipopolysaccharide (LPS), the major component of the outer membrane of Gram-negative bacteria, and of other bacterial metabolites (e.g., deoxycholic acid, DCA) have been described. Translocation of intestinal bacteria is also one of the most common complications of chronic liver disease, with increased systemic levels of bacterial components (pathogen-associated molecular patterns, PAMPs) due to increased intestinal permeability. This is one of the several factors that also lead to a low-grade chronic inflammatory state that may contribute to carcinogenesis. LPS acts via activation of Toll-like receptor (TLR) 4, which has been shown to promote HCC development in animal models [70] by enhancing proliferative and antiapoptotic signals in chronically damaged liver cells. Moreover, the increased enterohepatic circulation of DCA causes functional changes in hepatic stellate cells, which adopt a senescence-associated secretory phenotype (SASP) [71] due to chronic stress (DCA is a known DNA damaging agent through ROS production) [72]. This condition promotes inflammation through the release of inflammatory cytokines, chemokines, and proteases (e.g., IL-1, IL-6, IL-8, CXCL9, PAI-1) but apparently not liver fibrosis [73]. In this way, SASP induces epithelial-mesenchymal transition and/or acquisition of features of malignancy by a paracrine mechanism. SASP in liver HSCs has been observed in areas of HCC in patients with NASH [74]. As a possible confirmation of these pathogenetic theories, observations of reduced tumor growth in intestinal sterilization by antibiotic therapies or lowering of DCA levels have been made in animal models [75].

It has also been observed that NAFLD tends to have a more aggressive fibrotic phenotype in patients with a history of appendectomy [76], so this may represent further evidence of the importance of gut homeostasis in the pathogenesis and progression of NAFLD/NASH.

Genetic polymorphisms might also contribute to the development of HCC in patients with NAFLD/NASH. For example, the I148M sequence variant of patatin-like phospholipase-3 (*PNPLA3*) is one of the strongest genetic determinants of NAFLD/NASH and is associated with increased fat deposition in the liver and hepatic inflammation. It has been shown to be an independent risk factor for HCC in patients with cirrhosis [77], especially in patients with NASH or alcoholic liver disease (OR 1.67). Other data report even stronger associations of this variant with NASH-HCC (OR 2.26 in heterozygosity, OR 5.00 in homozygosity) [78]. 

## 4. Clinical Impact of Different Pathogenetic Drivers in the Management of HCC

NAFLD-NASH is currently estimated to affect 25% of the global population and is associated with an increased risk of HCC [79]. Metabolic risk factors commonly associated with NAFLD, including diabetes mellitus type II, obesity, and metabolic syndrome, are thus becoming emerging risk factors for HCC. Clinical management of NAFLD should consist of the treatment of liver disease and associated metabolic comorbidities and conditions. Furthermore, there is also a strong association between NAFLD, increased risk of cardiovascular (CV) events, and mortality; therefore, the control of CV risk factors is essential. Patients with NAFLD without NASH have an excellent prognosis and pharmacological treatments with the objective of improving liver disease are generally limited to patients with NASH and biopsy-confirmed fibrosis. The most effective treatment for NAFLD seems to be a lifestyle-improving intervention: weight loss results in histopathology modifications, decreasing portal inflammation and fibrosis. Interestingly, low-calorie diet and aerobic exercise programs have also been associated with reduced liver fat and lowered CV risk [80]. Finally, some trials have observed that statins significantly improve aminotransferase values and CV outcomes in patients with elevated ALT/AST concentrations, therefore lipid-lowering therapy is considered a standard of care for patients with NAFLD [81].

Indeed, statins have proved to be important tools against liver fibrosis, portal hypertension and HCC chemoprevention, exerting their effects through multiple pleiotropic mechanisms [82]. In addition to their lipid-lowering effects, statins also have anti-inflammatory, antioxidant and antithrombotic activity, which are particularly important for NAFLD/NASH, in which oxidative stress and inflammation play a major role.

Therefore, statins are widely recommended in available guidelines to tackle the dyslipidemia and increased CV morbidity and mortality associated with both NAFLD and NASH [81], in both primary and secondary prevention. 

Moreover, a Cochrane meta-analysis [83] concluded that statins may improve aminotransferase levels and imaging findings in patients with NAFLD, but data on NASH histology improvements are insufficient to draw a conclusion. Although more evidence from adequately powered trials is needed to prove the efficacy of statins for the treatment of NAFLD and its hepatic and extrahepatic complications, epidemiological and experimental studies have identified statins as one of the few available candidates for chemoprevention of HCC [82,84]. 

### NAFLD and HCC Treatment

HCC is the second leading cause of cancer-related deaths in the world. The prognosis of patients with HCC remains poor, with a median five-year survival around 18% [85]. Its intrinsic resistance to chemotherapy is known, however HCC treatment has seen important innovations since 2008 (Table 1). 

Currently, the main approved treatments for advanced HCC are multikinase inhibitors, anti-VEGF-R2 antibodies and combinations with immunotherapy. Despite the recognized role of NASH and NAFLD in the pathogenesis of HCC, outcomes of patients harboring these conditions are not fully described in the pivotal studies conducted so far and unfortunately were not taken into account in stratification strategies. Sorafenib was the first tyrosine kinase inhibitor (TKI) to receive Food and Drug Administration approval for systemic treatment of HCC and has been the standard first-line therapy for unresectable HCC since 2007. Indeed, the SHARP trial, which enrolled 602 patients, demonstrated a 2.8 month overall survival (OS) improvement in favor of sorafenib versus placebo (HR 0.69; 95% Cl, 0.55–0.87; *p* < 0.001) [86]. Later, in 2018, 954 eligible patients were randomly assigned to lenvatinib (*n* = 478) or sorafenib (*n* = 476) in the non-inferiority phase III trial REFLECT, which demonstrated the non-inferiority of lenvatinib as first-line treatment compared with sorafenib, with 13.6 months of median survival time for lenvatinib, and 12.3 months for sorafenib (HR 0.92, 95% CI 0.79–1.06, *p* value not significant). Notably, the objective response rate (ORR) for lenvatinib was higher (24% vs. 9% OR 3.13, 95% CI 2.15–4.56; *p* <  0.0001) and the progression free survival (PFS) was longer (7.4 vs. 3.7 months, HR 0.66, 95% CI 0.57–0.77, *p* < 0.0001) compared to sorafenib, thus making lenvatinib the new standard of care [87]. 

Additionally, in the phase III study Imbrave-150, the efficacy of the combination of atezolizumab and bevacizumab versus sorafenib was investigated. The intention-to-treat population included 336 patients in the atezolizumab–bevacizumab group and 165 patients in the sorafenib group. The combination treatment demonstrated increased OS and PFS. Indeed, at the time of the primary analysis, 12-months OS rate was 67.2% in the experimental arm (95% CI 61.3–73.1) and 54.6% in the control arm (95% CI 45.2–64.0), while median PFS (mPFS) was 6.8 months and 4.3 months respectively (HR 0.59, 95% CI 0.47–0.76; *p* < 0.001). Among the study population, about 30% of patients who received atezolizumab plus bevacizumab had a non-viral etiology, however, the number of cases diagnosed with NAFLD/NASH was not reported [88]. The association of pembrolizumab and lenvatinib also demonstrated antitumor activity and good safety in patients with unresectable HCC in a phase Ib trial. Efficacy and safety were assessed in 104 patients, with an ORR of 46% (95% CI 36.0–56.3%) per mRECIST, mPFS was 9.3 months (95% CI 5.6–9.7 months) per mRECIST and 8.6 months per RECIST v1.1 (95% CI 7.1–9.7 months). Median reported OS was 22.0 months (95% CI 20.4 months to NE) [89], leading to additional clinical trial phases. In another trial, nivolumab was tested versus sorafenib as first line treatment in patients with HCC and the study was presented as an abstract at ESMO 2020. In this study, nivolumab showed clinically meaningful improvements in OS, ORR, and complete response rate as first line treatment. At a median follow-up of 33.6 months, mOS was 16.4 months vs 14.8 months, HR 0.85, 95% CI 0.72–1.00, *p* = 0.0522 and the 33-month OS rate was 29% for nivolumab and 21% for sorafenib. Overall, 45% of patients treated with nivolumab had a non-viral etiology, while 31% and 21% had HBV or HCV infection, respectively. Consistent OS benefit with nivolumab was observed regardless of PD-L1 status or viral etiology [90]. Unfortunately, in the pivotal treatment trials with sorafenib [86], lenvatinib [87] and lenvatinib-pembrolizumab [89], no data about NAFLD were reported. 

Second line treatment following first line failure has been investigated in several international clinical trials. Indeed, the phase III RESORCE trial showed that regorafenib, at the dose of 160 mg once daily for the first 3 weeks of each 4-week cycle, increased OS compared to placebo. Overall, 843 patients were screened, of whom 573 were enrolled and randomized (379 to regorafenib and 194 to placebo). Median reported OS was 10.6 months versus 7.8 months (HR 0.63, 95% CI 0.50–0.79; one-sided *p* < 0.0001) for regorafenib and placebo groups respectively, in patients with HCC who had progressed to previous sorafenib treatment. PFS in patients treated with regorafenib was 3.1 months versus 1.5 months (HR 0.46, 95% CI 0.37–0.56; one-sided *p* < 0.0001), Disease control rate (DCR) was 66% in the regorafenib group (one-sided *p* < 0.0001) with 54% of patients experiencing stable disease. Thirty-eight patients out of 573 (25 in regorafenib arm and 13 in controlled arm respectively) had a history of NASH and the main risk factor reported for HCC was HBV infection (38% of patients in each arm) [91]. A post-hoc analysis of the RESORCE trial suggested that in patients progressed to sorafenib, regorafenib conferred a similar clinical benefit regardless of last drug dose or time to progression to sorafenib [92].

Additionally, the efficacy of cabozantinib in pretreated advanced HCC was demonstrated in the phase III CELESTIAL trial. In this study, which enrolled 707 patients, cabozantinib increased the mOS compared to placebo from 8.0 months to 10.2 months (HR 0.76, 95% CI 0.63–0.92; *p* = 0.005) [93]. The difference was higher in patients who had only received sorafenib. Median PFS was also higher with cabozantinib, 5.2 months compared to 1.9 months with placebo (HR 0.44, 95% CI 0.36–0.52; *p* < 0.001). Overall, 43 of 470 (9%) patients in the experimental arm and 23 of 237 (10%) in the control arm showed NASH, while the most common etiology in both arms was HBV infection. In the subgroup analyses, patients who had most benefit from cabozantinib were those with HBV-related HCC. 

More recently, pembrolizumab received accelerated approval in patients with advanced HCC in the second line setting, based on the preliminary results of the phase II trial Keynote-224. Activity and safety were assessed in 104 patients with an ORR of 18.3% (similar across subgroups), a mPFS of 4.9 months, a mOS of 13.2 months and a 24-months rate PFS and OS of 11.3% and 30.8%, respectively [94]. Drawing from these data, the Keynote-240 trial evaluated the efficacy and safety of pembrolizumab in 413 patients, but did not reach prespecified statistical significance, although an improvement in OS and PFS was reported, supporting a favorable risk-to-benefit ratio for pembrolizumab (mOS 13.9 months in pembrolizumab-treated patients versus 10.6 months in placebo-treated patients, HR 0.78, 95% CI 0.611–0.998, *p* = 0.023; mPFS 3.0 versus 2.8 months, respectively, HR 0.718, 95% CI 0.570–0.904, *p* = 0.0022). Notably, in this trial 59% of patients had no history of HBV-HCV infection [95].

In all the trials previously reported, adverse events (AEs) were not distinguished according to HCC etiology and no clinical data are currently available on treatment-induced AEs in subgroups with NAFLD-related HCC. The most common grade 3-4 AEs were similar in the different pivotal trials. In the SHARP study the most common were diarrhea (8%), hand–foot syndrome (8%), hypertension (2%), and abdominal pain (2%) [86]; in the RESORCE trial, hypertension (15%), hand–foot syndrome (13%), fatigue (9%) and diarrhea (3%) [91], while in the CELESTIAL trial palmar-plantar erythrodysesthesia (16%), hypertension (16%) and increased aspartate aminotransferase (12%) [93]. Grade 3–4 AEs correlated to lenvatinib in the REFLECT trial also were hypertension (23%), palmar-plantar erythrodysesthesia (3%) and diarrhea (4%) [87], thus confirming a class effect of multikinase inhibitors on adverse events. In Keynote-240, incidence of any treatment-related grade 3 or higher AEs was 52.7% and 46.3% in the pembrolizumab and placebo group, respectively, and the most frequent was the increase in AST levels (13.3%) [95]. Finally, in Keynote 524 [90] and Imbrave 150 [88], the most common grade 3–4 treatment-related AEs were hypertension (17% and 15% respectively) and increased AST (11% and 7% respectively).

A recent meta-analysis of three large randomized controlled phase III trials analyzed the effects of immunotherapy in patients with advanced HCC. It was observed that, although immunotherapy improved survival in the overall population and in all the subgroups, improvement was greater in HBV- and HCV-related cases, suggesting a role of viral infection in inducing immunotherapy susceptibility [96]. These findings, considering the heterogeneity in disease etiology and treatment setting, provide limited evidence that needs further confirmations. However, these data suggest how a non-viral etiology of liver damage, such as NAFLD and NASH, could be a predictor of unfavorable outcomes in patients treated with immune-checkpoint inhibitors.

## 5. Discussion

Cirrhosis is a well-known precursor lesion and main risk factor for HCC onset. Nevertheless, a variable proportion of HCC cases can occur in absence of cirrhosis, although there is a lack of systematic studies investigating the etiological profile of HCC in non-cirrhotic liver. Among major risk factors for this condition, NAFLD and HBV are the most studied since they are known to be directly involved in liver mutagenesis. Given that most strategies for preventing HCC, such as surveillance, involve individuals with evident cirrhosis, a better understanding of the development of HCC in the absence of cirrhosis is needed because of its potential implications on current clinical practice. In fact, about 20–30% of HCC cases in NAFLD patients occur in absence of cirrhotic liver [19,97]. Therefore, as cirrhosis does not appear to be necessary for HCC onset, it has been hypothesized that obesity, insulin resistance and the pro-inflammatory microenvironment of NAFLD can directly mediate carcinogenesis in this subset of patients. Although precise pathogenesis has not been clearly defined yet, several pathways could play a major role in the setting of NAFLD without cirrhosis, including excessive oxidative stress, activation of the unfolded protein response and the innate immune system [2]. All these events can, indeed, lead to DNA damage, providing a favorable setting for the development of HCC.

NAFLD is currently one of the most common liver diseases in Western countries [98]. The burden of NAFLD is primarily driven by the prevalence of obesity and type 2 diabetes and therefore it is expected to gradually increase over time. Nevertheless, diagnostic strategies and treatment options that specifically focus on NAFLD remain very limited. NAFLD is also among the most represented risk factors for HCC; therefore, a better knowledge of HCC in NAFLD with or without cirrhosis is urgent due to its clinical impact. No specific recommendations are available for NAFLD-related HCC, its staging and treatment still remain based on the Barcelona Clinic Liver Cancer (BCLC) staging. Regarding clinical trials, few studies stratified HCC patients by risk factors for the underlying liver disease, although it is an important variable affecting disease clinical course. 

NAFLD-related HCC has been mainly categorized as “non-viral” or “from other causes” within most clinical studies, with the exception of REFLECT and CELESTIAL trials, in which the subgroup of NASH has been reported in the descriptive analysis [87,93]. Therefore, prospective randomized trials that more specifically take into account the under-represented group of NAFLD-related HCC patients are currently needed. A better knowledge of molecular and clinical aspects of NAFLD and associated HCC could pave the way to individualized prevention, surveillance and treatment strategies. Moreover, the overlap between clinical manifestations of metabolic syndrome characterizing NAFLD and commonly observed adverse events make clinical management of these patients even more difficult and this enhances the urgent need of specific evidence, with a proper stratification according to the etiology underpinning liver injury, in order to better define the best candidates to receive each available treatment.

In conclusion, NAFLD represents a modern challenge in the management of HCC patients and, despite its growing incidence, a strong effort is still needed to better refine its clinical implications.

## Figures and Tables

**Table 1:** Overview on main clinical trials on advanced HCC.

Clinical Trials	Outcomes (Experimental Arm vs. Control Arm)	Non-Viral Etiology (Experimental Arm vs. Control Arm)
**SHARP**: sorafenib vs. placebo (1st line) NCT00105443	**OS**: 10.7 vs. 7.9 months; HR 0.69, 95% CI, 0.55–0.87, *p* < 0.001 **PR**: 2% vs. 1% **SD**: 71% vs. 67% 1-yr survival rate: 44% vs. 33%	Alcoholic 26% vs. 26% Other 9% vs. 10% Unknown 16% vs. 19%
**REFLECT**: lenvatinib vs sorafenib (1st line) ^a^ NCT01761266	**OS**: 13.6 vs. 12.3 months; HR 0.92, 95% CI 0.79–1.06, p not significant **PFS**: 7.4 vs. 3.7 months; HR 0.66, 95% CI 0.57−0.77, *p* < 0.0001 **ORR**: 24.1% vs. 9.2%; OR 3.13, 95% CI 2.15–4.56, *p* < 0.0001	Alcoholic 8% vs. 4% Other 8% vs. 7% Unknown 13% vs. 14%
**IMBRAVE 150**: atezolizumab-bevacizumab vs sorafenib (1st line) ^b^ NCT03434379	**OS at 6 months**: 84.8% (95% CI 80.9–88.7) vs. 72.2% (95% CI 65.1–79.4) **OS at 12 months**: 67.2% (95% CI 61.3–73.1) vs. 54.6% (95% CI, 45.2–64.0) **PFS**: 6.8 vs. 4.3 months; HR 0.59, 95% CI, 0.47–0.76, *p* < 0.001 **ORR**: 27.3% vs. 11.9%, *p* < 0.001	Non-viral 30% vs. 32%
**KEYNOTE 524**: lenvatinib-pembrolizumab (1st line) ^a^ NCT03006926	**OS**: 22.0 months (95% CI, 20.4 months-NE) **PFS**: 8.2 months (95% CI, 7.4–9.7 months) **ORR**: 46.0% (95% CI, 36.0–56.3%)	Alcoholic 28% Other 22%
**CHECKMATE 459**: nivolumab-sorafenib (1st line) ^b^ NCT02576509	**OS**: 16.4 vs. 14.8 months; HR 0.85, 95% CI 0.72–1.00, *p* = 0.0522	Non-viral 45% vs. 45%
**RESORCE**: regorafenib vs placebo (2nd line) ^a^ NCT01774344	**OS**: 10.6 vs. 7.8 months; HR 0.63, 95% CI 0.50–0.79, *p* < 0.0001 **PFS**: 3.1 vs. 1.5 months; HR 0.46, 95% CI 0.37–0.56), *p* < 0.0001 **ORR**: 11% vs. 4%, *p* = 0.0047	Alcoholic 24% vs. 28% NASH 7% vs. 7% Other 7% vs. 5% Unknown 17% vs. 16%
**CELESTIAL**: cabozantinib vs. placebo (2nd line) ^b^ NCT01908426	**OS**: 10.2 vs. 8.0 months; HR 0.76, 95% CI 0.63–0.92, *p* = 0.005 **PFS**: 5.2 vs. 1.9 months; HR 0.44, 95% CI 0.36–0.52, *p* < 0.001 **ORR**: 4% vs. <1%, *p* = 0.009	Alcoholic 24% vs. 16% NASH 9% vs. 10% Other 5% vs. 7% Unknown 16% vs. 20%
**KEYNOTE 240**: pembrolizumab vs. placebo (2nd line) ^b^ NCT02702401	**OS**: 13.9 vs. 10.6 months; HR 0.78, 95% CI 0.611–0.998; *p* = 0.0238 **PFS**: 3.0 vs. 2.8 months; HR 0.718, 95% CI 0.570–0.904, *p* = 0.0022 **ORR**: 18.3% vs. 4.4%, *p* = 0.00007	Non-viral 58.6% vs. 63.0%

^a^ assessed according to mRECIST. ^b^ assessed according to RECIST (version 1.1). Abbreviations: OS, Overall Survival; PFS, Progression Free Survival; ORR, Objective Response Rate; PR, Partial Response; DS, Stable Disease; NASH, non-alcoholic steato-hepatitis.

## Data Availability

Not applicable.
